# Predictors of Mortality in Adults Admitted with COVID-19: Retrospective Cohort Study from New York City

**DOI:** 10.5811/westjem.2020.6.47919

**Published:** 2020-07-08

**Authors:** Sridhar Chilimuri, Haozhe Sun, Ahmed Alemam, Nikhitha Mantri, Elona Shehi, Jairo Tejada, Alla Yugay, Suresh K. Nayudu

**Affiliations:** BronxCare Health System, Department of Medicine, Affiliated with Icahn School of Medicine at Mount Sinai, Bronx, New York

## Abstract

**Introduction:**

Rapid spread of coronavirus disease 2019 (COVID-19) in the United States, especially in New York City (NYC), led to a tremendous increase in hospitalizations and mortality. There is very limited data available that associates outcomes during hospitalization in patients with COVID-19.

**Methods:**

In this retrospective cohort study, we reviewed the health records of patients with COVID-19 who were admitted from March 9–April 9, 2020, to a community hospital in NYC. Subjects with confirmed reverse transcriptase-polymerase chain reaction (RT-PCR) of the nasopharyngeal swab for severe acute respiratory syndrome coronavirus-2 (SARS-CoV-2) were included. We collected data related to demographics, laboratory results, and outcome of hospitalization. Outcome was measured based on whether the patient was discharged home or died during hospitalization.

**Results:**

There were 888 consecutive admissions with COVID-19 during the study period, of which 513 were excluded with pending outcome or incomplete information. We included a total of 375 patients in the study, of whom 215 (57%) survived and 160 (43%) died during hospitalization. The majority of patients were male (63%) and of Hispanic origin (66%) followed by Blacks (25%), and others (9%). Hypertension (60%) stands out to be the most common comorbidity followed by diabetes mellitus (47%), cardiovascular disease (17%), chronic kidney disease (17%), and human immunodeficiency virus/acquired immunodeficiency syndrome (9%). On multiple regression analysis, increasing odds of mortality during hospitalization was associated with older age (odds ratio [OR] 1.04; 95% confidence interval [CI], 1.01–1.06 per year increase; p < 0.0001), admission D-dimer more than 1000 nanograms per milliliter (ng/mL) (OR 3.16; 95% CI, 1.75–5.73; p<0.0001), admission C-reactive protein (CRP) levels of more than 200 milligrams per liter (mg/L) (OR 2.43; 95% CI, 1.36–4.34; p = 0.0028), and admission lymphopenia (OR 2.63; CI, 1.47–4.69; p 0.0010).

**Conclusion:**

In this retrospective cohort study originating in NYC, older age, admission levels of D-dimer of more than 1000 ng/mL, CRP of more than 200 mg/L and lymphopenia were associated with mortality in individuals hospitalized for COVID-19. We recommend using these risk factors on admission to triage patients to critical care units or surge units to maximize the use of surge capacity beds.

## INTRODUCTION

Coronavirus disease 2019 (COVID-19) is a viral infectious disease caused by severe acute respiratory syndrome coronavirus 2 (SARS-CoV-2). SARS-CoV-2 belongs to the coronaviridae family of viruses, with (SARS-CoV) and Middle East respiratory syndrome coronavirus being other members of the same family.^1^ COVID-19 started as a cluster of unknown pneumonia cases in Wuhan, China, in December 2019.^2^ The World Health Organization declared COVID-19 a pandemic on March 11, 2020.^3^ Since then the number of positive cases has increased exponentially, spreading to scores of countries including the United States.^4^

New York state, especially New York City (NYC) and surrounding boroughs, has experienced the highest infection rate of COVID-19 in the US, leading to significant morbidity and mortality. As of April 12, 2020, there have been more than 110,000 cases diagnosed with 30,000 hospitalizations in NYC. Hospitalizations associated with COVID-19 in NYC have led to significant challenges in human and infrastructure resource allocation for healthcare institutions.^5^ There are few reports related to resource allocation and triaging the patients admitted to hospitals.^6^,^7^ In the middle of the pandemic we attempted to evaluate our experience in search of any clinical and/or laboratory predictors that would help us to rapidly triage patients to appropriate units.

The borough of the Bronx has a population with a poverty rate double that of the national average.^8^ Healthcare dynamics in this area are complex^9^ due to prevailing socioeconomic and cultural challenges in the community.^10^ The Bronxcare Health System (BCHS) hospital in the South Bronx serves this population, which was faced with one of the highest infection rates of COVID-19 in the US.

## METHODS

### Study Design

We conducted this retrospective cohort study at BCHS, a safety-net hospital located in the Bronx, New York, US. The study was approved by the institutional review board (IRB) at BCHS, and written informed consent was waived by the IRB owing to the observational nature of the study in a rapidly evolving pandemic. The chart abstractors were blinded to the study hypothesis.

### Participants and Eligibility Criteria

We retrospectively analyzed consecutive patients who had been admitted to our hospital between March 9, 2020 and April 9, 2020 and who were diagnosed as having COVID-19. Individuals aged 18 years and above were included in the study. Diagnosis of COVID-19 was defined as the patient having a positive result on the nasopharyngeal swab for SARS-CoV-2 by reverse transcriptase polymerase chain reaction (RT-PCR). Nasopharyngeal swab samples were collected at the time of admission, and testing was performed by RT-PCR assay. The hospital used test kits from several manufacturers, made available by the New York State Department of Health (DOH) during surge phase.

**Population Health Research Capsule**

What do we already know about this issue?

*Coronavirus disease 2019 (COVID-19) has been associated with significant mortality. Few reports identify risk factors for mortality in patients hospitalized with COVID-19*.

What was the research question?

What are the risk factors for mortality in hospitalized population with COVID-19 infection?

What was the major finding of the study?

*Increased odds of mortality were noted with older age, and admission values of D-dimer, C-reactive protein, and lymphopenia*.

How does this improve population health?

*Use of these indicators upon admission may help to triage COVID-19 patients in a surge capacity situation*.

All patients were admitted to the hospital through the emergency department (ED). Laboratory and radiological tests were performed based on clinical care needs of patients following existing medical and critical care guidelines. Laboratory tests included complete blood count, coagulation profile, liver panel, basic metabolic panel, C-reactive protein (CRP), lactate dehydrogenase (LDH), and other tests as indicated. Common radiological assessment included chest radiograph and computed tomography of the chest based on clinical decision-making. Patients were managed with supportive care and specific pharmacological protocols created by the hospital’s COVID-19 management guidelines committee in accordance with the Centers for Diseases Control and Prevention and the NY State DOH. Specific pharmacological treatments included systemic corticosteroids, hydroxychloroquine, colchicine, antiretroviral medications, and Tocilizumab.

A total of 888 patients with laboratory-confirmed SARS-CoV-2 were admitted during the study period to BCHS hospital. We excluded from the final analysis patients who were still receiving care in the hospital at the time of preparation of this manuscript and those patients with incomplete information

### Data Collection

We reviewed electronic health records, nursing records, and laboratory findings of all patients with laboratory-confirmed SARS-CoV-2 infection. Demographic data such as age, gender, and ethnicity were extracted. We also collected information on comorbid conditions including hypertension (HTN), diabetes mellitus (DM), obstructive airway disease (OAD), cardiovascular disease (CVD), chronic kidney disease (CKD), end stage renal disease, and human immunodeficiency syndrome (HIV)/acquired immunodeficiency syndrome (AIDS). Details of hospital course, use of ventilators, and laboratory results were also collected. We divided patients into two groups for final analysis based on survival at the end of hospital course (discharged vs deceased). Collected data were cross-checked by the authors at the end of data collection. Any disagreement between two authors was resolved by consulting with all authors and reaching consensus agreement.

### Statistical Analysis

We performed all statistical analyses using JMP 14, Mac version (SAS, Cary, NC).^11^ Continuous variable was expressed as median with interquartile range (IQR). Categorical variables were represented as counts and percentages. We used Fisher’s exact test to compare nominal variables between two groups, and we compared continuous variables using an independent, two-tailed t-test. Relation between risk factors and in-hospital mortality was measured using univariable and multivariable regression. We excluded variables from the univariate analysis if the difference was not significant or number of variables was very small. A two-sided ∝ value of less than 0.05 was considered statistically significant.

## RESULTS

A total of 888 consecutive patients were hospitalized in BCHS hospital with COVID-19 between March 9, 2020 and April 9, 2020. In the final analysis, we excluded the following patients: those whose SARS-Cov-2 results were pending or whose definitive outcomes were not available at the time of the study as they were still hospitalized; and those with incomplete information. We excluded 513 patients and included a total of 375 in the final analysis.

Of 375 patients, 215 (57%) were discharged home safely and 160 (43%) died during hospitalization. Median age was 63 years (range 19–97, interquartile range [IQR] 52.0–72.0). The majority of the hospitalized patients were male (63%) with male-to-female ratio of 12:7. Ethnic distribution of the study population was as follows: Hispanic (66%); Black (25%); and other (9%) (Table 1). This ethnic distribution differs from the surrounding Bronx community where Hispanics and Blacks represent 54.4% and 43.6% of the population, respectively.^8^ Comorbid conditions were present in three out of every four patients (77%) with HTN being the most common (60%) followed by DM (47%), CVD (17%) OAD (17%), CKD (14%), HIV/AIDS (9%), and chronic liver disease, (5%). Admission laboratory findings showed neutrophilia (26%), neutropenia (2%), lymphopenia (62%), and lymphocytosis (1%). Baseline characteristics of the study patients are shown in Table 1. Out of the 375 patients, 320 (85%) received hydroxychloroquine; 9 (2%) received antiretroviral medications; 12 (3%) received colchicine; and 3 (1%) received tocilizumab. Median time from hospitalization to outcome (discharge or death) was five days (IQR 3–8 days).

Male gender (70%), HTN (72%), DM (56%), CVD (24%), CKD (19%) and HIV/AIDS (9%) were noted with high prevalence in the deceased group. We also noted that neutrophilia was more frequent in the deceased group compared to the discharged group (34% vs 19%, p = 0.0077). Lymphopenia was predominant in the deceased group with 122 patients (76% vs 51%, p <0.0001) compared to survivors. Admission LDH, CRP, D-dimer, and ferritin levels were higher in the deceased group compared to the survivor group (Table 1).

On multiple regression analyses (Table 2), we observed increasing odds of mortality during hospitalization associated with older age (odds ratio [OR] 1.04, 95% confidence interval [CI], 1.01–1.06 per year increase, p = 0.0001), admission D-dimer levels of more than 1000 nanogram/milliliter (ng/mL) (OR 3.16; 95% CI 1.75–5.73; p<0.0001) admission CRP levels of more than 200 milligrams/liter (mg/L) (OR 2.43; 95% CI, 1.36–4.34; p = 0.0028) and admission lymphopenia (OR 2.63 [1.47–4.69]; p = 0.0028). Mean time from hospital admission to discharge was five days (IQR 3.0–8.0) and to death was five days (IQR 2.3–8.0; p = 0.91). There were more Hispanics admitted compared to Blacks (66% vs 25%) with a similar trend observed in the deceased (68% vs 24%) and survived groups (64% vs. 25%) (Table 1).

## DISCUSSION

The rapid, ongoing COVID-19 pandemic resulted in an exponential increase in the number of infected individuals in New York, especially NYC. As of April 15, 2020, approximately 30,000 people were hospitalized leading to an enormous burden on the NYC healthcare system and its providers.^5^ This impact was more significant in safety-net hospitals. Very limited data is available to determine risk factors and their association with outcomes in COVID-19 patients, to help hospitals and providers in triaging and managing these patients more efficiently.^12^–^15^ Considering a higher surge of cases in densely populated cities such as NYC triaging tools would enable the appropriate allocation of resources.

We looked at several risk factors in hospitalized adults with COVID-19 in this study. The higher death (43%) rate in our study may reflect the first two weeks of the epidemic and the lack of data on final outcomes on currently hospitalized patients. Our patients had a higher burden of underlying medical conditions, in particular HTN,^14^,^16^ which is expected in urban populations.

Odds of death during hospitalization were higher with increased age, which is in accordance with findings in other recent studies.^17^ Additionally, we found three important admission laboratory markers – D-dimer, CRP levels, and lymphopenia – to be useful in predicting outcomes (Table 3). Elevated D-dimer may represent alteration of the coagulation cascade including development of severe microembolic disease. Microembolic disease appears to be a major contributor of death in COVID-19 patients.

Triage models built on these risk factors would assist in allocation of resources and managing patients in appropriate critical care or modified units.

## LIMITATIONS

Our study has certain limitations as the excluded patients were still in the hospital with continuing clinical care at the time of preparation of this manuscript. Therefore, impact of outcome of these individuals is not currently known. The majority of our patients were Hispanics and Blacks, constituting more than 90% of the total study population. Thus, we did not make any conclusions regarding association between ethnicity and outcome due to very minimal representation from other ethnic groups.

## CONCLUSION

Our findings suggest that older age, admission D-dimer (>1000 ng/mL), CRP (>200 mg/lL), and lymphopenia (1.0 x10^3^/microliter) provide a reliable panel of tools to evaluate patients hospitalized with COVID-19 (Table 3). Our study is the only one to report lymphopenia and its association to mortality, as prior studies in this regard were inconclusive.^18^,^19^ In a surge, EDs need tools to appropriately triage patients and maximize utilization of critical care beds. Although our study population mainly represents Hispanics and Blacks, we believe results could be applied to all ethnic groups.

## Figures and Tables

**Table 1:** Baseline characteristics of patients diagnosed with coronavirus disease 2019 (COVID-19) after hospital admission.

	Total (N = 375)	Deceased (N = 160)	Survived (N = 215)	P-value
Demographic and clinical characteristics				
Age, years	63.0 (52.0–72.0)	68.0 (60.0–75.0)	58.0 (48.0–68.0)	< 0.0001
Gender				0.0173
Female	139 (37%)	48 (30%)	91 (42%)	
Male	236 (63%)	112 (70%)	124 (58%)	
Ethnicity				0.4553
Black	93 (25%)	39 (24%)	54 (25%)	
Hispanic	246 (66%)	109 (68%)	137 (64%)	
Other	36 (9%)	12 (8%)	24 (11%)	
Comorbidity	287 (77%)	142 (89%)	145 (67%)	<0.0001
Hypertension	225 (60%)	115 (72%)	110 (51%)	< 0.0001
Diabetes	175 (47%)	90 (56%)	85 40%)	0.0017
Cardiovascular disease	62 (17%)	38 (24%)	24 (11%)	0.0018
Obstructive airway disease	62 (17%)	29 (18%)	33 (15%)	0.4854
Chronic kidney disease	51 (14%)	31 (19%)	20 (9%)	0.0060
HIV/AIDS	22 (6%)	14 (9%)	8 (4%)	0.0469
Chronic liver disease	18 (5%)	11 (7%)	7 (3%)	0.1420
Laboratory markers (at the time of admission)				
Neutrophil count (NC) (x10^3^/microliter)	5.9 (4.0–8.2)	6.25 (4.3–8.8)	5.3 (3.8–7.5)	0.0393
<1.5 x10^3^/microliter	7 (2%)	3 (2%)	4 (2%)	0.0077
1.5–8.0 x10^3^/microliter	272 (72%)	103 (64%)	169 (79%)	0.0077
>8.0 x10^3^/microliter	96 (26%)	54 (34%)	42 (19%)	0.0077
Lymphocyte count (LC) (x10^3^/microliter)	0.8 (0.6–1.2)	0.7 (0.5–0.9)	0.9 (0.7–1.3)	0.8738
<1.0x10^3^/microliter	232 (62%)	122 (76%)	110 (51%)	<0.0001
1.0–4.8 x10^3^/microliter	139 (37%)	36 (23%)	103 (48%)	<0.0001
>4.8 x10^3^/microliter	4 (1%)	2 (1%)	2 (1%)	<0.0001
(NC/LC ratio)	6.9 (4.1–11.0))	8.75 (5.13–13.77)	6.0 (3.5–8.8)	< 0.0001
Lactate dehydrogenase (LDH) (unit/liter)	483.0 (341.0–700.0)	561.0 (426.0–800.0)	416.0 (297.0–598.0)	< 0.0001
C reactive protein (CRP) (milligram/liter)	122.2 (64.4–209.0)	160.0 (88.0–260)	97.0 (50.0–171.0)	< 0.0001
D-dimer (nanogram/milliliter)	504 (296.0–1010.0)	831.0 (408.0–2297.0)	394.0 (268.0–677)	< 0.0001
Ferritin (nanogram/milliliter)	820.0 (377.0–1511.0)	987.0 (490.0–1932.0)	717.0 (356.0–1379)	0.0026

*HIV*, human immunodeficiency virus; *AIDS*, acquired immunodeficiency syndrome.

**Table 2:** Multivariable analysis of baseline characteristics of hospitalized patients diagnosed with coronavirus disease 2019 (COVID-19).

	Univariable OR (95% CI)	P-value	Multivariable OR (95% CI)	P-value
Demographic and clinical characteristics				
Age, years	1.05 (1.03–1.73)	<0.0001	1.04 (1.01–1.06)	0.0003
Male gender (vs female)	1.71 (1.11–2.64)	0.0149	1.37 (0.79–2.37)	0.2520
Comorbidity present (vs not present)				
Hypertension	2.43 (1.57–3.77)	<0.0001	1.46 (0.82–2.62)	0.2046
Diabetes	1.96 (1.29–2.98)	0.0014	1.58 (0.94–2.65)	0.0841
Cardiovascular disease	2.48 (1.42–4.38)	0.0012	1.56 (0.78–3.11)	0.2025
Obstructive airway disease	1.22 (0.71–2.11)	0.47		
Chronic kidney disease	2.34 (1.28–4.29)	0.0058	1.33 (0.63–2.77)	0.3039
HIV/AIDS	2.48(1.02–6.07)	0.0464		
Chronic liver disease	2.19 (0.83–5.79)	0.1127		
Laboratory markers (at the time of admission)				
Absolute neutrophil count (ANC) (x10^3^/microliter)				
<1.5 x10^3^/microliter	1.23 (0.12–2.75)	0.4956	1.75 (0.31–9.95)	0.5273
1.5–8.0 x10^3^/microliter	1 (ref)			
>8.0 x10^3^/microliter	2.11 (1.32–3.38)	0.0019	1.57 (0.82–2.99)	0.1646
Absolute lymphocyte count (ALC) (x103/microliter)				
<1.0x10^3^/microliter	3.17 (2.0–5.02)	<0.001	2.63 (1.47–4.69)	0.0010
1.0–4.8 x10^3^/microliter	1 (ref)			
>4.8 x10^3^/microliter	2.86 (0.39–21.06)	0.3021	5.69 (0.69–46.9)	0.1056
(ANC/ALC) ratio (>11.0)	1.58 (1.03–1.09)	<0.0001	0.75 (0.37–1.53)	0.4385
Lactate dehydrogenase (LDH) (> 700 unit/liter)	2.51 (1.55–4.07)	0.0002	1.43 (0.79–2.59)	0.2357
C reactive protein (CRP) (> 200 milligram/liter)	2.85 (1.78–4.57)	<0.0001	2.43 (1.36–4.34)	0.0028
D-dimer (> 1000 nanogram/milliliter)	4.62 (2.79–7.63)	<0.0001	3.16 (1.75–5.73)	<0.0001
Ferritin (nanogram/milliliter)	1.87 (1.16–3.00)	0.0092	1.58 (0.89–2.80)	0.1183

*OR*, odds ratio; *CI*, confidence interval; *HIV*, human immunodeficiency virus; *AIDS*, acquired immunodeficiency syndrome.

**Table 3:** Key demographic and laboratory factors associated with mortality from COVID-19.

Elderly age (OR 1.04 for every one year increase; 95% CI, 1.001–1.06; p = 0.0003) Admission D-dimer level >1000 nanograms/milliliter (OR 3.16; 95% 1.75–5.73; p<0.0001) Admission CRP level >200milligrams/liter (OR 2.43; 95% CI, 1.36–4.34; p = 0.0028) Admission lymphopenia (<1.0x10^3^/microliter, OR 2.63; 95% CI, 1.47–4.69; p = 0.0010)

*OR*, odds ratio; *CRP*, C Reactive Protein, *CI*, confidence interval.
